# PEDIATRIX SAFE KIDS: Serious Game on the safety of pediatric patients

**DOI:** 10.1590/1980-220X-REEUSP-2024-0382en

**Published:** 2025-05-12

**Authors:** Léia Arcanjo Mendes, Clarissa Costa Antunes, Maíra Ferreira Sant’Ana, Anna Caroline Leite Costa, Francis Solange Vieira Tourinho, Bruna Figueiredo Manzo

**Affiliations:** 1 Universidade Federal de Minas Gerais, Escola de Enfermagem, Belo Horizonte, MG, Brazil.; 2 Universidade Federal de Minas Gerais, Departamento de Letras, Belo Horizonte, MG, Brazil.; 3 Universidade Federal de Minas Gerais, Hospital das Clínicas, Escola de Enfermagem, Belo Horizonte, MG, Brazil.; 4 Empresa Brasileira de Serviços Hospitalares, Belo Horizonte, MG, Brazil.; 5 Universitair Medisch Centrum Groningen, Groningen, The Netherlands.; 6 Universidade Federal de Santa Catarina, Florianópolis, SC, Brazil.; 7 Universidade Federal de Minas Gerais, Escola de Enfermagem, Enfermagem materno infantil e saúde pública, Belo Horizonte, MG, Brazil.

**Keywords:** Patient Safety, Digital Health, Mobile Applications, Games, Experimental, Educational Technology, Child

## Abstract

**Objective:**

To create and validate a mobile application of Serious Game to support the education of companions about pediatric patient safety actions.

**Method:**

Technological study with a framework based on Instructional Design. The application content was developed based on the findings of focus groups carried out with the companions of pediatric patients in October 2023, and theoretical contribution that included the international patient safety goals and scientific evidence. Judges validated the content between October 2023 and January 2024.

**Results:**

The mobile application of Serious Game in the format of quiz was called “Pediatrix safe kids” and has characters and icons. The final version of the mobile application contains 102 question screens, grouped into seven sections, with random access. The content validation stage took place with the 14 judges in two rounds, which presented an IVC > 0.80.

**Conclusion:**

The mobile application “Pediatrix safe kids” can help in the learning process of pediatric companions in a playful way in search for safe care.

## INTRODUCTION

Playfulness is a strategy to achieve the teaching and learning process by promoting creativity, reflection, integration, and behavioral change^(1)^. Among the models of recreational activities, there are the Serious Games (SG)^(2)^. Unlike traditional games, SGs are designed not only to entertain, but also to teach and train, aiming to promote significant behavioral changes^(1,2,3)^. They promote interactive experiences that engage and motivate users to acquire new knowledge and skills^(3)^. These games have been applied in the health area as a teaching and education strategy^(3)^, so that their use can contribute to promoting family members’ learning about patient care^(4)^.

In addition to traditional forms, such as board games, SG can be made available digitally, such as digital health games, being qualified as Digital Health (DH)^(4)^. DH is a comprehensive field, which integrates Information and Communication Technologies and aims at access and quality of health services^(5)^. Among the technologies that make up DH are mobile applications, which are software programmed for mobile devices^(6)^. Scientific evidence indicates that the use of these applications in healthcare settings promotes knowledge among users, engagement in proposed actions^(7)^, and the promotion of patient safety (PS)^(4)^.

PS aims to reduce risks and prevent adverse events (AE), with a focus on continuous improvement of care processes, being considered one of the fundamental pillars to guarantee care quality^(8)^. The PS theme has been discussed since the beginning of the millennium and its scope is still a global challenge^(8)^. Despite advances in implementing practices to improve safety, such as protocols standardization, adverse events (AE) indicators remain a critical concern^(8)^. In the pediatric context, the situation is even more alarming because, despite measures to improve safety, adverse event rates are still high and require the adoption of new strategies^(9)^. A Japanese study indicated that 37% of pediatric patients presented at least one AE during the period of hospitalization in the wards^(9)^. In this scenario, patients’ companions can play a critical role as a protective factor, helping to reduce the occurrence of errors and contributing to safety in the hospital environment^(4)^.

Effective strategies to mitigate errors include education and active participation of caregivers in the care process, as evidenced by the Patient and Family Centered Care model, which encourages the inclusion of the family in care^(10)^. Although companions are willing to participate, they often lack the necessary knowledge to contribute effectively to PS^(4)^. The lack of understanding of patients’ companions about safety protocols, such as care for hand hygiene, correct administration of medication, and recognition of warning signs in children, can lead to failures in actions to prevent AEs^(4)^. Therefore, it is essential that educational strategies are implemented to provide caregivers with the tools and knowledge required for them to play their role more effectively in promoting PS^(4)^.

In this context, considering the availability of mobile applications and the evident need to invest in strategies that promote education about pediatric PS for children’s companions, the need to create and validate a mobile application of Serious Game about the safety of the pediatric patient, whose target audience is their companions, was observed. This application will be used in hospital pediatric units, where companions play a crucial role, given that they spend most of their time with the child. Therefore, the objective of the present study is to create and validate a mobile application of Serious Game to support the education of companions about pediatric patient safety actions.

## METHOD

### Design of Study

This is a technological study based on the methodological framework of Instructional Design (ID), known by the acronym ADDIE^(11)^. The ADDIE model is divided into the phases of A-analysis, D-design, D-development, I-implementation and A-evaluation, which occur in an integrated manner^(11)^. This study presents an excerpt from the process, covering part of the Analysis phase, in addition to the Design, Development, and Evaluation phases of the mobile application, with validation by specialist professionals.

The creation of the SG mobile application was also based on the Conceptual Model by Garret, which considers the user experience as a central axis, proposing that their demands and goals are understood and considered in the process^(12)^. This model helps ensure that the application not only works well, but also provides an enjoyable experience, which is essential to the effectiveness of an SG in health education and engagement.

Given the importance of the user’s experience, before starting to create SG content, focus groups were held with the companions of pediatric patients who were in the admission unit. These groups aimed to diagnose prior knowledge and information gaps of companions of pediatric patients regarding patient safety.

In the SG created, the content was based on the results of the focus groups and the International Patient Safety Goals (IPSG)^(13)^. Additionally, national and international manuals and resolutions were also used to support the creation of content. Furthermore, the results of the scoping review previously carried out and a technological prospecting^(14)^ contributed to the foundation of the content. This research meets the recommendations of the Standards for Quality Improvement Reporting Excellence (SQUIRE 2.0), as it is an approach to improving health care.

After surveying the items that would make up the mobile application of SG, the Naming, design and programming project was performed. In the SG and brand design stage, it was decided that an accessible tool would be created, considering that the target audience is mostly female, between 18 and 60 years old, and with varying socioeconomic conditions. Moreover, with the aim of developing an innovative, unique brand with a young and confident personality, the design stage was guided by keywords such as: “Fun”, “Safety”, “Curious”, “Human”, “Playful” and “Trustworthy”. These words were chosen with the purpose that the brand’s design and communication would reflect an engaging and safe experience, aligned with the public’s values and expectations.

The application programming used the Visual Studio Code (VS Code) as a code editor. As for the language used, React-native and Javascript were adopted, allowing the application to be developed natively to Apple Store and Google Play. During the development of the application, the content underwent a language evaluation process, accomplished by a collaborator in the field of linguistics.

### Period

The mobile application of SG “Pediatrix Safe Kids” was created and validated in the period from October 2021 to February 2024.

### Participants

The focus groups were carried out with the companions of patients who were in a pediatric inpatient unit of a Municipal Public Hospital in Belo Horizonte, which has capacity for 35 beds. The pediatric hospitalization unit was considered to be the sectors providing care to patients between 29 days and 13 years of age, considering the public served in this hospital and based on the Statute of the Child and Adolescent (ECA). The neonatal population was excluded, as they have specific language and characteristics. For this step, companions of patients who had been hospitalized for more than 24 hours and who understood the Brazilian Portuguese were selected. Companions who were under 18 years of age or who had some hearing or visual impairment were excluded. The sample was for convenience and the patients’ companions were invited at the patient’s bedside, individually.

The mobile application was created in partnership with the company Specter Labs’s information technology team and a professional in the field of linguistics. Thus, the characters, icons and all questions are original and authorial.

In the content validation stage, judges participated, whose role was to assess whether the items of the developed tool referred to the phenomenon of interest that would be evaluated^(15)^. Therefore, content validation by the judges aimed to ensure that the information contained in the mobile application created in this work were accurate, scientific, up-to-date, and relevant, taking into account the best practices, guidelines, and evidence in the area of pediatric patient safety. The group of judges consisted of nursing professionals who were specialized in pediatrics, or nursing professionals with clinical practice of at least two years in pediatrics, or with a master’s or doctorate degree with dissertation r thesis in the PS area in the pediatric context or who had research in the pediatric context.

The analysis of the judges’ CVs was carried out intentionally using the Lattes Platform, considering the keywords “PS”; “Pediatrics”, the filter for the Nursing area and the databases: PhDs and other researchers of Brazilian nationality.

Subsequently, 84 judges from all over the country were invited to the 1st round of validation of the application’s content. Invitations were sent via the Lattes Platform, email or multiplatform instant messaging application and voice calls to smartphones. In a complementary way, the snowball technique was also applied. This technique consists of an approach in which the first research participants indicate other judges, considering the eligibility criteria^(16)^.

### Instruments used for Data Collection

The focus groups were conducted based on 29 open questions, which were previously prepared by the researchers. The questions were categorized according to the general theme of PS and the IPSG. The group audios were recorded in digital format.

To validate the application’s content, an instrument was constructed in the form of a questionnaire, using the online research management application. This instrument was built based on 12 evaluation criteria, namely: behavioral, objectivity, simplicity, clarity, relevance, precision, variety, modality, typicality, credibility, amplitude, and balance^(15)^.

### Data Collection

The focus groups were carried out by two previously trained researchers who were not employees of the institution where the research took place. Each focus group lasted an average of 40 minutes. After the explanations about the research, clarification of doubts and signing of the Free and Informed Consent Form (FICF), the groups began with a brief introduction, whose aim was to reassure and establish the framework, and then a presentation of the participants was made. Then the group was conducted.

In the content validation stage, the judges had access to digital material, which contained instructions on the content validation process and the FICF. This material also includes a link to access the electronic form (Google Forms), where the instrument could be found with the questions for content validation. Initially, a 20-day deadline to respond to the form was established, which was later extended to 1 month.

### Data Analysis

After collection, the contents of the focus groups were transcribed and checked by 02 researchers separately for information accuracy and precision. Then they were typed on Microsoft Office Word 2007®. These contents were grouped according to the similarity of the theme. During transcription, participants’ names were replaced with “Companion’s number”.

The analysis of the information obtained in the focus groups with the companions was conducted using content and theme techniques. As a support tool, the “MaxQDA” software was used. The content and theme analysis seeks to interpret communications through the identification and categorization of evaluations^(17)^.

Content validation applied the technique Delphi in the data collection stage, with them being considered valid if carried out from 02 to 04 rounds^(18)^. Furthermore, this collection was carried out with the Likert scale, in which the score varies from 1 to 5, with 1 corresponding to totally agree, 2 agree, 3 neither agree nor disagree, 4 disagree, and 5 totally disagree^(19)^.

The analysis of the judges’ opinion data considered the content validity index (CVI). The calculation of the CVI is performed by dividing the number of responses 4 or 5 by the total number of responses obtained with the application of the instrument^(20)^. The reference agreement value among judges corresponds to values above 0.80^(21)^. Ideally, this value is expected to be greater than 0.90^(21)^.

### Ethical Aspects of Research

This study followed the ethical aspects of research involving human beings, according to Resolutions 466/2012, 510/2016 and 674/2022 of the National Health Council (CNS).

The research project was submitted and approved by the Human Research Ethics Committees of the Universidade Federal de Minas Gerais, with opinion number 5.547.309.

The data from this study are in the possession of the principal researcher and may be made available upon request.

## RESULTS

The mobile application “Pediatrix Safe Kids” was created between October 2021 and February 2024. The first version was released on digital platforms Apple Store® and Google Play® in September 2023. Its content considered prior knowledge and the main doubts and demands of the companions of patients who were identified in the focus groups, as well as the theoretical contribution, which includes the international patient safety goals and evidence from the scientific literature.

The focus groups were held in October 2023, with the participation of 19 companions of pediatric patients. These patients were between 0 and 7 years old (average 2.9 years) and were hospitalized for a period ranging from 1 to 49 days, with a median of 9 days. As for the focus group participants, the majority (17, or 89.4%) were female, aged between 21 and 42 years (average of 31.9 years). Regarding education, 11 (57.9%) finished high school, 3 (15.8%) finished elementary school, 1 (5.3%) had an undergraduate degree, 1 (5.3%) was illiterate, and 3 (15.7%) did not provide information. Everyone had a cell phone, and 18 (94.7%) used mobile applications such as WhatsApp and Instagram.

In the focus groups, when asked whether the companions of pediatric patients had received guidance on PS in the past year, 12 (63.2%) responded yes and seven (36.8%) said no. During the focus groups, the companions demonstrated a lack of clarity about the concept of PS. In addition, situations emerged that were indicated to be considered when creating the mobile application, such as information on the use of antibiotics, the presence of a companion during procedures and post-discharge care. The importance of clear and effective communication was also highlighted.

**Figure 1:**
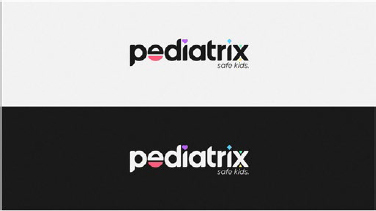
Brand signature “Pediatrix Safe Kids”. Belo Horizonte, MG, Brazil, 2022.

Source: Prepared by the company Specter Labs exclusively, the brand “Pediatrix safe kids”, 2022.

Concurrently with the focus group stage, the process of creating the brand “Pediatrix Safe Kids” began, including the brand colorimetry process (Figure 1), which was based on the colors used in the PS promotion campaign by the Ministry of Health.

The brand “Pediatrix Safe Kids” aims to unite knowledge, accessibility, and technology. Therefore, the creation of the application screens aimed to promote knowledge among the pediatric patient’s companion through the presentation of situations that were similar to those experienced in pediatric hospitalization units, which require reflection and intervention and failure prevention actions.

The first action of this brand was the development of a SG mobile application in the format of a quiz in the modality single player. This quiz has 90 questions, which were distributed in six blocks. Each block represents one of the IPSG and has 15 questions. In these blocks, each question has four answer options, of which only one is correct. Moreover, the SG “Pediatrix Safe Kids” has 12 challenge questions, with two questions for each IPSG. Each challenge question has two answer options, with only one of these options being correct. Challenge questions appear randomly throughout the game.

**Figure 2:**
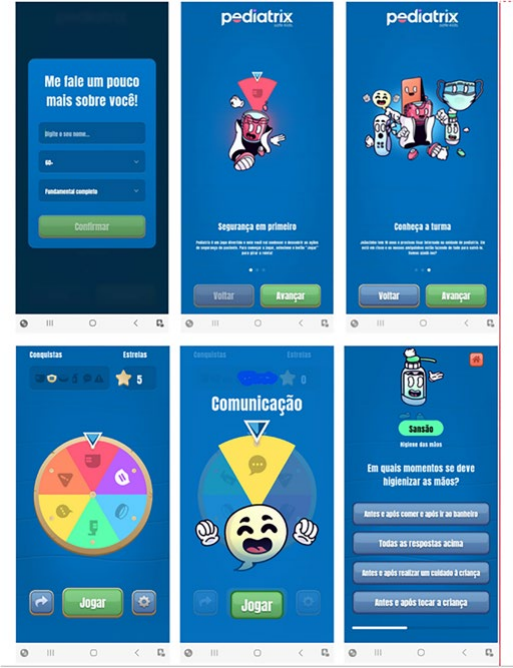
“Pediatrix Safe Kids” SG mobile application screens. Belo Horizonte, MG, Brazil, 2022.

Source: Prepared by the author, 2023.

**Figure 3:**
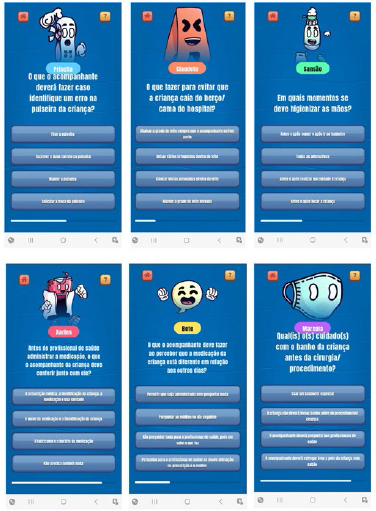
Questions Contained in the “Pediatrix Safe Kids” App Quiz. Belo Horizonte, MG, Brazil, 2022.

Source: Prepared by the author, 2023.

**Figure 4:**
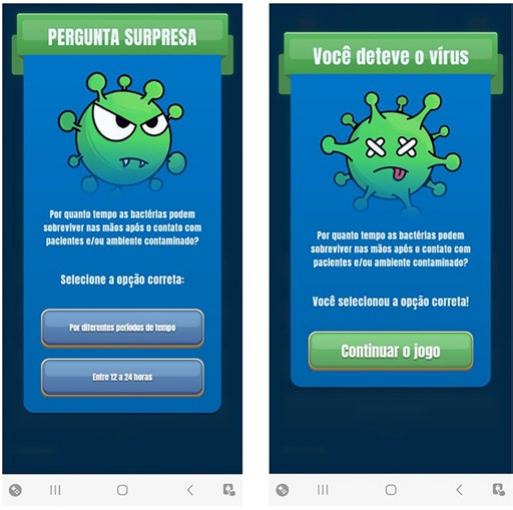
Example of a Challenge Question Contained in the “Pediatrix Safe Kids” App Quiz. Belo. Belo Horizonte, MG, Brasil, 2022.

Source: Prepared by the author, 2023.

The initial SG screens are: 1st player presentation (first name, age and education); 2nd up to the 4th screen is the SG presentation, which contains instructions on how to play; 5th to 11th present the characters (Figure 2). Afterwards, the player will select the option to play and spin the roulette wheel, which will randomly select a block of questions related to one of the IPSG (Figures 3 and 4). By completing all the questions in a goal, the player will earn an icon and their stars will increase. However, if the player leaves the SG without completing the selected goal, the questions that were answered in this goal will be lost. At the end of each goal, the player returns to the roulette screen. Upon completion of the SG, the player will be taken to the celebration screen. Afterwards, he will choose to save his achievement or clear his achievements and restart SG. The question screens have a button to present the theoretical framework considered for constructing the question and respective answer. In addition, in every question, the player receives a feedback, no which highlights whether his/her answer was right or wrong.

**Figure 5:**
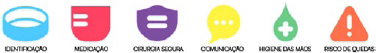
Icons representing the International PS Goals present in the brand “Pediatrix Safe Kids”. Belo Horizonte, MG, Brasil, 2022.

Source: Prepared by the company Specter Labs exclusively, the brand “Pediatrix safe kids”, 2022.

A set of icons (Figure 5) has been created to enrich the user experience in SG “Pediatrix Safe Kids”.

**Figure 6:**
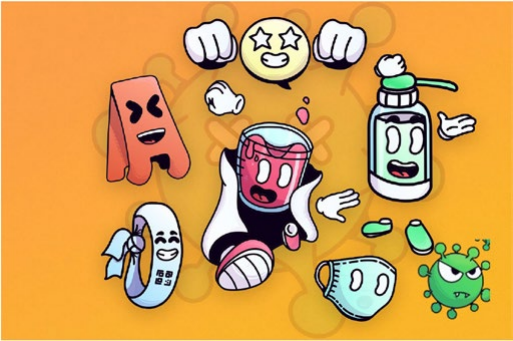
Characters of the Game “Pediatrix Safe Kids”. Belo Horizonte, MG, Brasil, 2022.

Source: Prepared by the company Specter Labs exclusively, the brand “Pediatrix safe kids”, 2022.

Furthermore, seven characters were created to compose the game (Figure 6), with the aim of helping to understand the SG content and bringing the mobile application closer to its target audience. This way, each of the six IPSG is represented by a character, just as the questions in the challenge block have a character to represent them.

The first version of the characters created was presented to companions and patients. Participants reported liking the characters; however, one of them did not represent the idea initially proposed for him, so he was edited.

The SG “Pediatrix Safe Kids” was created to be interactive and generate entertainment, allowing continuity and interaction across multiple sessions, enabling, for example, the target audience to finish the game and have the opportunity to play it again. Thus, this SG was not created to be completed in a single session, which can last approximately an hour and a half.

The validation of SG content with specialist professionals took place in two rounds Delphi, in the period from October 2023 to January 2024. In the 1st round, 14 judges agreed to participate in the research, with the states of residence being Santa Catarina, Minas Gerais, Espírito Santo, Bahia, and Rio Grande do Norte.

The participants’ age ranged from 26 to 65 years, with a median of 38 years. The time of work in pediatrics ranged from one to 20 years, with a median of eight and a half years. Among the participants, eight (57.1%) responded that they completed their degree more than 10 years ago. Regarding academic qualifications, six (42.9%) had a master’s degree and six (42.9%) had a doctorate. Regarding the area of professional activity (teaching, research, assistance and management), eight (57.2%) specialist professionals indicated that they worked in two or more areas.

In the first round of the “Pediatrix Safe Kids” application content evaluation process, some expert professionals suggested editing the SG presentation content, highlighting the presentation of characters in the first screens, improving the identification of each of the IPSG throughout the SG, and editing some questions, as well as some answer options so that the message would be clearer. After the necessary editions and based on the evaluation of specialist professionals, the content of the SG went through second round of validation. At this stage, all items reached an IVC above 0.80. Therefore, all content reached suitability levels.

## DISCUSSION

Companions of pediatric patients act as a barrier in the process of preventing adverse events when they engage in PS actions^(4)^. For this engagement to occur, education on PS, with a focus on sharing information and responsibilities, is necessary^(22)^. Therefore, SGs are a strategy for the education of companions of pediatric patients, considering the provision of information, which also helps in understanding shared responsibilities^(22)^.

Scientific literature has been shown to be favorable to the use of digital technologies, including applications, as effective tools to promote health education, especially in pediatric settings^(23)^.

A documentary research study on mobile applications and their contributions to caregivers in the context of child development demonstrated that these tools allow care guidance on issues that affect childhood development^(23)^. Another study found that the use of a mobile application on patient safety aimed at the pediatric public resulted in an increase in children’s awareness levels about safety incidents at the hospital level^(24)^.

Considering access to information, SGs can be made available in mobile applications for smartphones, which favors access to information in different geographic regions and at any time^(6)^. A survey carried out in Brazil, with 13,360 participants, found that 73.9% had the habit of playing digital games, with the the smartphone being the preferred platform for playing for 48.8% of players^(25)^.

Therefore, the SG “Pediatrix Safe Kids”, through mobile application technology, emerges as an strategy for educating companions of pediatric patients regarding PS, taking into account the accessibility to information, which can promote engagement in the proposed PS actions.

SG is an educational tool that can be created and developed for use by different audiences^(7)^. The process of creating a tool involves concern for meeting the needs and providing a satisfactory experience to the target audience of this product^(12)^. For this to occur, it must get closer to the target audience since the beginning of the process, seeking to identify their prior knowledge, their demands, as well as the characteristics of this public and the resources they already have^(26)^. This knowledge makes the final product to have a language that is appropriate for the target audience, the information to be current and relevant, and the scenarios to correspond to the real world^(26)^. With this as a reference, the mobile application “Pediatrix Safe Kids” content was based on the results of the focus groups conducted in the present study.

Knowledge about the target audience of a tool also helps in the process of creating the brand, which goes beyond functionalities^(12)^. The visual aspects of the brand, such as colors and typography, can generate associations or emotional reactions, thus becoming communication strategies and approaching the target audience^(12)^. Accordingly, the application “Pediatrix Safe Kids” was created based on an identity study, besides considering the objectives proposed with its use. In addition, other tools can be added to the application later, aiming to increase accessibility to information. Among these tools is the audio with questions and answer options and character animations.

The creation of the “Pediatrix Safe Kids” also aimed to provide quality, precise content, and to be a relevant strategy for the promotion of PS. Therefore, content validation was carried out with specialist professionals. Thus, with the aim of standardizing the language for different languages and cultures and uniting different knowledge about pediatric PS^(27)^, judges from all over the country were invited.

In the first round of validation, the judges found weaknesses in the application’s content that were related to the production of results that are independent of the subjectivity of the tool’s user, to the expression of a single idea that does not confuse the user, and to the assessment of whether a given content is sufficient to cover the entire magnitude of a theme, which represents the amplitude^(15)^. These points were edited to provide visual and logical application facilitation, so that the proposed message can be understood^(28)^. Consequently, in the second round of the method Delphi, the content of the mobile application SG “Pediatrix Safe Kids” has been validated and can therefore be applied to support the education of companions regarding pediatric PS.

Thus, it is considered that the application can be used to contribute to the health area and the results of this research should be disseminated to collaborate with future studies related to the advancement and use of technologies aimed at health education in the long term.

As a limitation of this study, we can mention the high cost of application updates on the digital platforms Google Play and Apple Store, which was minimized in this work through obtaining funding.

## CONCLUSION

The creation of the SG mobile application was based on a model centered on the user’s experience, using different research methods. The content of this application has been defined based on the IPGS and international scientific evidence. At the end, the content was validated by professionals who were specialists in pediatric care or in the PS area.

It is an innovative educational tool, which has significant potential to support education of companions of pediatric patients regarding PS. Therefore, an increase in access to information with scientific validity is expected and we hope to contribute to the reduction of adverse events in pediatrics.

The validation process with the target audience should be continued, considering mainly the usability validation of the mobile application.
