# Supplementary material for: PEDIATRIX SAFE KIDS: Serious Game on the safety of pediatric patients
Source: Rev Esc Enferm USP. 2025 May 12;59:e20240382. doi: 10.1590/1980-220X-REEUSP-2024-0382en (PMC12097365; doi:10.1590/1980-220X-REEUSP-2024-0382en)
Supplement: Translation

# Translation: PEDIATRIX SAFE KIDS: Serious Game sobre a segurança dos pacientes pediátricos

**DOI:** 10.1590/1980-220X-REEUSP-2024-0382pt

**Authors:** Léia Arcanjo Mendes, Clarissa Costa Antunes, Maíra Ferreira Sant’Ana, Anna Caroline Leite Costa, Francis Solange Vieira Tourinho, Bruna Figueiredo Manzo

**Affiliations:** 1 Universidade Federal de Minas Gerais, Escola de Enfermagem, Belo Horizonte, MG, Brasil.; 2 Universidade Federal de Minas Gerais, Departamento de Letras, Belo Horizonte, MG, Brasil.; 3 Universidade Federal de Minas Gerais, Hospital das Clínicas, Escola de Enfermagem, Belo Horizonte, MG, Brasil.; 4 Empresa Brasileira de Serviços Hospitalares, Belo Horizonte, MG, Brasil.; 5 Universitair Medisch Centrum Groningen, Groningen, The Netherlands.; 6 Universidade Federal de Santa Catarina, Florianópolis, SC, Brasil.; 7 Universidade Federal de Minas Gerais, Escola de Enfermagem, Enfermagem materno infantil e saúde pública, Belo Horizonte, MG, Brasil.

**Keywords:** Segurança do Paciente, Saúde Digital, Aplicativos Móveis, Jogos Experimentais, Tecnologia Educacional, Criança

## Abstract

**Objetivo:**

Criar e validar um aplicativo móvel de *Serious Game* para o apoio à educação dos acompanhantes acerca das ações de segurança do paciente pediátrico.

**Método:**

Estudo tecnológico com referencial pautado no Design Instrucional. O conteúdo do aplicativo foi elaborado a partir dos achados de grupos focais realizados com os acompanhantes dos pacientes pediátricos em outubro de 2023, e aporte teórico que incluíram as metas internacionais de segurança do paciente e evidências científicas. Juízes validaram o conteúdo entre outubro de 2023 e janeiro de 2024.

**Resultados:**

O aplicativo móvel de *Serious Game* em formato de *quiz,* recebeu o nome *“Pediatrix safe kids*” e conta com personagens e ícones*.* A versão final do aplicativo móvel contém 102 telas de perguntas, agrupadas em sete seções, com acesso aleatório. A etapa de validação do conteúdo ocorreu com os 14 juízes em duas rodadas, as quais apresentaram um IVC > 0,80.

**Conclusão:**

O aplicativo móvel “*Pediatrix safe kids*” pode auxiliar no processo de aprendizagem dos acompanhantes da pediatria de maneira lúdica em busca do cuidado seguro.

## INTRODUÇÃO

O lúdico é uma estratégia para se alcançar o processo de ensino e aprendizado promovendo a criatividade, reflexão, integração e mudança de comportamento^(1)^. Entre os modelos de atividades lúdicas, têm-se os *Serious Game* (SG)^(2)^. Diferentemente dos jogos tradicionais, os SG são projetados não apenas para entreter, mas também para ensinar e capacitar, visando à promoção de mudanças comportamentais significativas^(1–3)^. Eles promovem experiências interativas que envolvem e motivam os usuários a adquirir novos conhecimentos e habilidades^(3)^. Esses jogos têm sido aplicados na área da saúde como estratégia de treinamento e de educação^(3)^, de modo que o seu uso pode contribuir para a promoção do aprendizado dos familiares sobre os cuidados com o paciente^(4)^.

Além das formas tradicionais, como os jogos de tabuleiros, os SG podem ser disponibilizados por meio digital, como os jogos digitais para a saúde, enquadrando-se como Saúde Digital (SD)^(4)^. A SD é um campo abrangente, o qual integra as Tecnologias da Informação e Comunicação e visa ao acesso e à qualidade dos serviços de saúde^(5)^. Dentre as tecnologias que compõem a SD estão os aplicativos móveis, que são softwares programados para dispositivos móveis^(6)^. Evidências científicas indicam que o uso desses aplicativos nos cenários de atenção à saúde promove o conhecimento dos seus usuários, o engajamento às ações propostas^(7)^ e a promoção da segurança do paciente (SP)^(4)^.

A SP visa à redução de riscos e à prevenção de eventos adversos (EA), com foco na melhoria contínua dos processos assistenciais, sendo considerada um dos pilares fundamentais para garantir a qualidade do cuidado^(8)^. A temática SP vem sendo discutida desde o início do milênio e o seu alcance ainda é um desafio global^(8)^. Apesar dos avanços na implementação de práticas para melhorar a segurança, como a padronização de protocolos, os indicadores de eventos adversos (EA) continuam a ser uma preocupação crítica^(8)^. No contexto pediátrico, a situação é ainda mais alarmante, pois, apesar das medidas para melhorar a segurança, os índices de eventos adversos ainda são elevados e demandam a adoção de novas estratégias^(9)^. Um estudo japonês indicou que 37% dos pacientes pediátricos sofreram pelo menos um EA durante o período de internação nas enfermarias^(9)^. Nesse cenário, os acompanhantes dos pacientes podem desempenhar um papel fundamental como fator protetor, ajudando a reduzir a ocorrência de erros e contribuindo para a segurança no ambiente hospitalar^(4)^.

Estratégias eficazes, para mitigar erros, incluem a educação e a participação ativa dos acompanhantes no processo de cuidado, como evidenciado pelo modelo de Cuidado Centrado no Paciente e na Família, que incentiva a inclusão da família no cuidado^(10)^. Embora os acompanhantes estejam dispostos a participar, frequentemente carecem do conhecimento necessário para contribuir efetivamente para a SP^(4)^. A falta de compreensão dos acompanhantes dos pacientes sobre os protocolos de segurança, como os cuidados com a higiene das mãos, a administração correta de medicamentos e o reconhecimento de sinais de alerta em crianças, pode levar a falhas nas ações de prevenção aos EA^(4)^. Dessa forma, é imprescindível que estratégias educativas sejam implementadas para fornecer aos acompanhantes as ferramentas e o conhecimento necessário para que desempenhem seu papel de forma mais eficaz na promoção da SP^(4)^.

Nesse contexto, considerando a disponibilidade dos aplicativos móveis e a evidente necessidade de se investir em estratégias que promovam a educação acerca da SP pediátrico para o acompanhante das crianças, observou-se a necessidade de criar e validar um aplicativo móvel de *Serious Game* sobre a segurança do paciente pediátrico, o qual tem como público alvo os seus acompanhantes. Este aplicativo será utilizado nas unidades pediátricas hospitalares, onde os acompanhantes desempenham um papel crucial, haja vista que passam a maior parte do tempo ao lado da criança. Sendo assim, o objetivo do presente estudo é criar e validar um aplicativo móvel de *Serious Game* para o apoio à educação dos acompanhantes acerca das ações de segurança do paciente pediátrico.

## MÉTODO

### Tipo de Estudo

Trata-se de um estudo tecnológico fundamentado no referencial metodológico do Design Instrucional (DI), conhecido pelo acrônimo ADDIE^(11)^. O modelo ADDIE divide-se nas fases de A-análise, D-desenho, D-desenvolvimento, I-implementação e A-avaliação, que ocorrem de forma integrada^(11)^. Este estudo apresenta um recorte do processo, abordando uma parte da fase de Análise, além das fases Desenho, Desenvolvimento e Avaliação do aplicativo móvel, com a validação por profissionais especialistas.

A criação do aplicativo móvel de SG fundamentou-se, também, no Modelo Conceitual de *Garret*, que considera a experiência do usuário como eixo central, propondo que suas demandas e seus objetivos sejam compreendidos e considerados no processo^(12)^. Esse modelo ajuda a assegurar que o aplicativo não apenas funcione bem, mas também proporcione uma experiência agradável, o que é essencial para a eficácia de um SG na educação e engajamento em saúde.

Dada a importância da experiência do usuário, antes de iniciar a criação do conteúdo do SG, foram realizados grupos focais com os acompanhantes dos pacientes pediátricos que estavam na unidade de internação. Esses grupos objetivaram o diagnóstico do conhecimento prévio e as lacunas de informação dos acompanhantes dos pacientes pediátricos quanto à segurança do paciente.

No SG criado, o conteúdo foi embasado nos resultados dos grupos focais e nas Metas Internacionais de Segurança do Paciente (IPSG)^(13)^. Adicionalmente, manuais e resoluções nacionais e internacionais também foram utilizados como apoio para a criação do conteúdo. Ademais, os resultados da revisão de escopo realizada previamente e uma prospecção tecnológica^(14)^ contribuíram para a fundamentação do conteúdo. Esta pesquisa atende às recomendações do *Standards for Quality Improvement Reporting Excellence* (SQUIRE 2.0), por se tratar de uma abordagem para melhoria nos cuidados de saúde.

Após realizar o levantamento dos itens que iriam compor o aplicativo móvel de *SG*, foi realizado o projeto de *Naming*, design e programação. Na etapa de concepção do SG e da marca, optou-se por criar uma ferramenta acessível considerando que o público-alvo seja majoritariamente feminino, está compreendido entre 18 a 60 anos, e com condições socioeconômicas variadas. Ainda, com o objetivo de desenvolver uma marca inovadora, ímpar e com personalidade jovem e confiante, a etapa de concepção foi orientada por palavras-chave como: “Divertida”, “Segurança”, “Curiosa”, “Humana”, “Lúdica” e “Confiável”. Essas palavras foram escolhidas com o propósito de que o design e a comunicação da marca refletissem uma experiência envolvente e segura, alinhada com os valores e as expectativas do público.

A programação do aplicativo utilizou o *Visual Studio Code (VS Code)* como editor de código. Quanto à linguagem utilizada, foi adotado o *React-native* e *Javascript,* permitindo que o aplicativo fosse desenvolvido nativamente para a *Apple Store* e *Google Play*. Durante a elaboração do aplicativo, o conteúdo passou por um processo de avaliação de linguagem, realizado por uma colaboradora da área da linguística.

### Período

O aplicativo móvel *de SG “Pediatrix Safe Kids”* foi criado e validado no período de outubro de 2021 a fevereiro de 2024.

### Participantes

Os grupos focais foram realizados com os acompanhantes dos pacientes que estavam em uma unidade de internação pediátrica de um Hospital Público Municipal de Belo Horizonte, o qual tem capacidade para 35 leitos. Considerou-se unidade de internação pediátrica os setores que prestam atendimento aos pacientes com idade entre 29 dias de vida até 13 anos, considerando o público atendido neste hospital e com base no Estatuto da Criança e do Adolescente (ECA). O público neonatal foi excluído, pois possui linguagem e características específicas. Para esta etapa, foram selecionados os acompanhantes de pacientes que estavam internados há mais de 24 horas e que compreendessem a língua portuguesa do Brasil. Foram excluídos os acompanhantes cuja idade era menor que 18 anos ou que apresentassem alguma deficiência auditiva ou visual. A amostra deu-se por conveniência e os acompanhantes dos pacientes foram convidados à beira leito do paciente, de forma individual.

O aplicativo móvel foi criado em parceria com profissionais da tecnologia da informação da empresa *Specter Labs* e um profissional da área da linguística. Desse modo, os personagens, os ícones e todas as perguntas são originais e autorais.

Na etapa de validação de conteúdo, participaram juízes, os quais tinham como função avaliar se os itens da ferramenta desenvolvida referia-se ou não ao fenômeno de interesse que seria avaliado^(15)^. Desse modo, a validação de conteúdo pelos juízes visou a assegurar que as informações contidas no aplicativo móvel criado no presente trabalho fossem precisas, científicas, atualizadas e de relevância, tendo em vista as melhores práticas, diretrizes e evidências da área de segurança do paciente pediátrico. O grupo de juízes foi composto por profissionais de enfermagem que eram especializados em pediatria, ou profissionais de enfermagem com prática clínica de, no mínimo, dois anos na pediatria, ou com titulação de mestre ou doutor com dissertação ou tese na área de SP no contexto pediátrico ou tivessem pesquisa no contexto pediátrico.

A análise do currículo dos juízes foi por meio da Plataforma Lattes, a qual ocorreu de forma intencional, considerando as palavras-chaves “SP”; “Pediatria”, o filtro para a área da Enfermagem e as bases: doutores e demais pesquisadores de nacionalidade brasileira.

Na sequência, foram convidados 84 juízes de todo o território nacional para a 1^a^ rodada de validação do conteúdo do aplicativo. Os convites foram realizados por meio da Plataforma Lattes, e-mail ou aplicativo multiplataforma de mensagens instantâneas e chamadas de voz para smartphones. De forma complementar, a técnica de Bola de Neve também foi aplicada. Essa técnica consiste em uma abordagem em que os primeiros participantes da pesquisa indicam outros juízes, considerando os critérios de elegibilidade^(16)^.

### Instrumentos Utilizados Para a Coleta de Dados

Os grupos focais foram conduzidos com base em 29 perguntas abertas, as quais foram elaboradas previamente pelas pesquisadoras. As perguntas foram categorizadas quanto à temática geral de SP e quanto às IPSG. Os áudios dos grupos foram gravados em formato digital.

Para a validação de conteúdo do aplicativo, foi construído um instrumento no formato de questionário, utilizando o aplicativo de gerenciamento de pesquisas online. Esse instrumento foi construído com base em 12 critérios de avaliação, sendo eles: comportamental, objetividade, simplicidade, clareza, relevância, precisão, variedade, modalidade, tipicidade, credibilidade, amplitude e equilíbrio^(15)^.

### Coleta de Dados

Os grupos focais foram realizados por 02 pesquisadoras treinadas previamente e que não eram funcionárias da instituição onde ocorreu a pesquisa. Cada grupo focal durou em média 40 minutos. Após as explicações acerca da pesquisa, esclarecimento de dúvidas e assinatura do Termo de Consentimento Livre e Esclarecido (TCLE), os grupos tiveram início com uma breve introdução, que tinha como objetivo tranquilizar e estabelecer o enquadre, e após foi realizada uma apresentação dos participantes. Em seguida, o grupo foi conduzido.

Já na etapa de validação do conteúdo, os juízes tiveram acesso a um material digital, o qual continha instruções sobre o processo de validação do conteúdo e o Termo de Consentimento Livre e Esclarecido (TCLE). Nesse material, foi incluído também o link de acesso ao formulário eletrônico (Google *Forms*), no qual estava o instrumento com as perguntas para a validação de conteúdo. Inicialmente, foi estabelecido um prazo de 20 dias para responder ao formulário, o qual, posteriormente, foi prorrogado para 01 mês.

### Análise dos Dados

Posteriormente à coleta, os conteúdos dos grupos focais foram transcritos e conferidos por 02 pesquisadoras separadamente, a fim de verificar a exatidão e precisão das informações. Em seguida, foram digitados no *Microsoft Office Word 2007* ^®^. Esses conteúdos foram agrupados conforme a semelhança da temática. Durante a transcrição, os nomes dos participantes foram substituídos por “Acompanhante ‘número’”.

A análise das informações obtidas nos grupos focais com os acompanhantes foi conduzida utilizando técnicas de conteúdo e temática. Como ferramenta de apoio, foi utilizado o software “MaxQDA”. A análise de conteúdo e temática busca a interpretação das comunicações por meio da identificação e categorização das avaliações^(17)^.

A validação de conteúdo aplicou a técnica *Delphi* na etapa de coleta dos dados, considerando como válido se realizado de 02 a 04 rodadas^(18)^. Ainda, essa coleta foi realizada por meio da aplicação da escala de *Likert*, na qual a pontuação varia entre 1 e 5, sendo que 1 corresponde a concordo totalmente, 2 concordo, 3 não concordo nem discordo, 4 discordo e 5 discordo totalmente^(19)^.

A análise dos dados de opinião dos juízes considerou o índice de validade de conteúdo (IVC). O cálculo do IVC é realizado de modo a dividir o número de respostas 4 ou 5 pelo número total de respostas obtidas com a aplicação do instrumento^(20)^. O valor de concordância de referência entre os juízes corresponde aos valores acima de 0,80^(21)^. Idealmente, espera-se que esse valor seja superior a 0,90^(21)^.

### Aspectos Éticos da Pesquisa

Este estudo respeitou os aspectos éticos da pesquisa envolvendo seres humanos, conforme as Resoluções 466/2012, 510/2016 e 674/2022 do Conselho Nacional de Saúde (CNS).

O projeto de pesquisa foi submetido e aprovado aos Comitês de Ética em Pesquisa com Seres Humanos da Universidade Federal de Minas Gerais, sob o parecer número nº 5.547.309.

Os dados deste estudo estão em posse da pesquisadora principal, podendo ser disponibilizados mediante solicitação.

## RESULTADOS

O aplicativo móvel “*Pediatrix Safe Kids*” foi criado entre outubro de 2021 e fevereiro de 2024. A primeira versão foi liberada nas plataformas digitais *Apple Store*® e *Google Play*® em setembro de 2023. Seu conteúdo considerou o conhecimento prévio e as principais dúvidas e demandas dos acompanhantes dos pacientes que foram identificados nos grupos focais, além do aporte teórico, o qual inclui as metas internacionais de segurança do paciente e evidências da literatura cientifica.

Os grupos focais foram realizados em outubro de 2023, com a participação de 19 acompanhantes de pacientes pediátricos. Esses pacientes tinham entre 0 a 7 anos (média 2,9 anos) e estavam internados por um período que variou de 1 a 49 dias, sendo a mediana de 9 dias. Quanto aos participantes do grupo focal, a maioria (17, ou 89,4%) era do gênero feminino, com idades entre 21 e 42 anos (média de 31,9 anos). Em relação à escolaridade, 11 (57,9%) concluíram até o ensino médio, 3 (15,8%) o ensino fundamental, 1 (5,3%) fez graduação, 1 (5,3%) era analfabeto e 3 (15,7%) não informaram. Todos possuíam celular, e 18 (94,7%) usavam aplicativos móveis como WhatsApp e Instagram.

Nos grupos focais, ao questionar se os acompanhantes do paciente pediátricos haviam recebido orientações sobre a SP no último ano, 12 (63,2%) responderam que sim e sete (36,8%) disseram que não. No decorrer dos grupos focais, os acompanhantes demonstraram falta de clareza sobre o conceito de SP. Além disso, emergiram situações que foram apontadas para serem consideradas na criação do aplicativo móvel, como informações sobre o uso de antibióticos, a presença do acompanhante em procedimentos e cuidados pós-alta. Também foi evidenciada a importância de uma comunicação clara e eficaz.

Concomitantemente à etapa do grupo focal, iniciou-se o processo de criação da marca “*Pediatrix Safe Kids*”, incluindo o processo de colorimetria da marca (Figura 1), a qual foi fundamentada nas cores utilizadas na campanha de promoção da SP pelo Ministério da Saúde.

**Figura 1:** Assinatura da marca “*Pediatrix Safe Kids*”. Belo Horizonte, MG, Brasil, 2022.

Fonte: Elaborado pela empresa *Specter Labs* exclusivamente a marca “*Pediatrix safe kids”*, 2022.

A marca “*Pediatrix Safe Kids*” busca unir conhecimento, acessibilidade e tecnologia. Sendo assim, a criação das telas do aplicativo visou à promoção do conhecimento do acompanhante do paciente pediátrico por meio da apresentação de situações semelhantes às vivenciadas nas unidades de internação pediátrica, as quais demandam reflexão e ações de intervenção e prevenção de falhas.

A primeira ação desta marca foi o desenvolvimento de um aplicativo móvel de SG em formato de *quiz* na modalidade *single player*. Este *quiz* é composto por 90 perguntas, distribuídas em seis blocos. Cada bloco representa uma das IPSG e possui 15 perguntas. Nesses blocos, cada pergunta possui quatro opções de resposta, sendo que apenas uma é a correta. Ainda, o SG “*Pediatrix Safe Kids*” possui 12 perguntas de desafio, sendo duas perguntas para cada IPSG. Cada pergunta de desafio possui duas opções de respostas, sendo que apenas uma destas opções é a correta. As perguntas desafios surgem aleatoriamente ao longo do jogo.

As telas iniciais do SG são: 1^a^ apresentação do jogador (primeiro nome, idade e escolaridade); da 2^a^ até 4^a^ tela é a apresentação do SG, que contém as instruções de como jogar; 5^a^ até a 11^a^ apresentam os personagens (Figura 2). Posteriormente, o jogador selecionará a opção de jogar e girará a roleta, que selecionará, de modo aleatório, um bloco de perguntas relacionado a uma das IPSG (Figuras 3 e 4). Ao completar todas as perguntas de uma meta, o jogador conquistará um ícone e suas estrelas irão aumentar. No entanto, caso o jogador saia do SG sem completar a meta selecionada, as perguntas que foram respondidas nesta meta serão perdidas. Ao final de cada meta, o jogador retorna à tela da roleta. Ao completar o SG, o jogador será encaminhado à tela de comemoração. Posteriormente, ele optará por salvar sua conquista ou limpar as suas conquistas e reiniciar o SG. As telas das perguntas possuem um botão de apresentação do referencial teórico considerado para a construção da pergunta e respectiva resposta. Ainda, em todas as perguntas, o jogador recebe um *feedback*, no qual é destacado se a sua resposta estava certa ou errada.

**Figura 2:** Telas do aplicativo móvel de SG “*Pediatrix Safe Kids*”. Belo Horizonte, MG, Brasil, 2022.

Fonte: Elaborado pela autora, 2023.

**Figura 3:** Perguntas Contidas no Quiz do Aplicativo “*Pediatrix Safe Kids*”. Belo Horizonte, MG, Brasil, 2022.

Fonte: Elaborado pela autora, 2023.

**Figura 4:** Exemplo de Pergunta-Desafio Contida no Quiz do Aplicativo “*Pediatrix Safe Kids*”. Belo Horizonte, MG, Brasil, 2022.

Fonte: Elaborado pela autora, 2023.

Um conjunto de ícones (Figura 5) foi criado para enriquecer a experiência do usuário no SG “*Pediatrix Safe Kids*”.

**Figura 5:** Ícones que representam as Metas Internacionais de SP presentes na marca “*Pediatrix Safe Kids*”. Belo Horizonte, MG, Brasil, 2022.

Fonte: Elaborado pela empresa *Specter Labs* exclusivamente a marca “*Pediatrix safe kids”*, 2022.

Ainda, para compor o jogo, foram criados sete personagens (Figura 6), com o objetivo de auxiliar na compreensão do conteúdo do SG e aproximar o aplicativo móvel do seu público-alvo. Desse modo, cada uma das seis IPSG é representada por um personagem, bem como as perguntas do bloco desafio possuem um personagem para representá-las.

**Figura 6:** Personagens do jogo “*Pediatrix Safe Kids*”. Belo Horizonte, MG, Brasil, 2022.

Fonte: Elaborado pela empresa *Specter Labs* exclusivamente a marca “*Pediatrix safe kids*”, 2022.

A primeira versão dos personagens criados foi apresentada aos acompanhantes e pacientes. Os participantes relataram gostar dos personagens, porém, um deles não representava a ideia inicialmente proposta para ele, de modo que ele foi editado.

O SG “*Pediatrix Safe Kids*” foi criado com o objetivo de ser interativo e de gerar entreterimento, de modo a possibilitar a continuidade e interação ao longo de múltiplas sessões, permitindo, por exemplo, que o público-alvo finalize o jogo e tenha a oportunidade de jogá-lo novamente. Desse modo, este SG não foi criado para ser concluído numa única sessão, que pode durar aproximadamente uma hora e meia.

A validação de conteúdo do SG com os profissionais especialistas ocorreu em duas rodadas *Delphi*, no período de outubro de 2023 a janeiro de 2024. Na 1^a^ rodada, 14 juízes aceitaram participar da pesquisa, sendo que os estados de residência foram Santa Catarina, Minas Gerais, Espírito Santo, Bahia e Rio Grande do Norte.

A idade das participantes variou de 26 a 65 anos, com mediana igual a 38 anos. O tempo de atuação na pediatria variou de um a 20 anos, com a mediana de oito anos e meio. Dentre as participantes, oito (57,1%) responderam que concluíram a graduação há mais de 10 anos. Em relação à titulação acadêmica, seis (42,9%) possuíam mestrado e seis (42,9%) possuíam doutorado. Quanto à área de atuação profissional (ensino, pesquisa, assistência e gestão), oito (57,2%) profissionais especialistas indicaram que atuavam em duas ou mais áreas.

Na primeira rodada do processo de avaliação do conteúdo do aplicativo “*Pediatrix Safe Kids*”, alguns profissionais especialistas sugeriram editar o conteúdo de apresentação do SG, destacar a apresentação dos personagens nas primeiras telas, melhorar a identificação de cada uma das IPSG ao longo do SG, e editar algumas perguntas, bem como algumas opções de resposta para que a mensagem ficasse mais clara. Após as edições necessárias e baseadas na avaliação dos profissionais especialistas, o conteúdo do SG passou pela segunda rodada de validação. Nesta etapa, todos os itens alcançaram um IVC acima de 0,80. Sendo assim, todo o conteúdo atingiu índices de adequabilidade.

## DISCUSSÃO

Os acompanhantes dos pacientes pediátricos atuam como barreira no processo de prevenção de eventos adversos quando se engajam nas ações de SP^(4)^. Para que esse engajamento ocorra, a educação em SP, com foco em compartilhar informações e responsabilidades, é necessária^(22)^. Sendo assim, os SG são uma estratégia para educação dos acompanhantes dos pacientes pediátricos considerando a disponibilização das informações, o que auxilia também na compreensão das responsabilidades compartilhadas^(22)^.

A literatura científica tem se mostrado favorável ao uso de tecnologias digitais, incluindo aplicativos, como ferramentas eficazes para promover a educação em saúde, especialmente em contextos pediátricos^(23)^. Um estudo de investigação documental acerca de aplicativos móveis e suas contribuições para os cuidadores no contexto do desenvolvimento infantil demonstrou que essas ferramentas permitem a orientação acerca do cuidado quanto a problemáticas que afetam o desenvolvimento na infância^(23)^. Um outro estudo identificou que o uso de um aplicativo móvel sobre a segurança do paciente voltado para o público pediátrico resultou em aumento dos níveis de conscientização das crianças sobre incidentes de segurança no nível hospitalar^(24)^.

Considerando o acesso às informações, os SG podem ser disponibilizados nos aplicativos móveis para *smarthphones* o que favorece o acesso à informação em diversas regiões geográficas e a qualquer momento^(6)^. Uma pesquisa realizada no Brasil, com 13360 participantes, identificou que 73,9% tinham hábito de jogar jogos digitais, sendo que a plataforma preferida para jogar de 48,8% dos jogadores era o *smarthphone* ^(25)^. Sendo assim, os SG “*Pediatrix Safe Kids*” por meio da tecnologia do aplicativo móvel emergem como estratégia para educação dos acompanhantes dos pacientes pediátricos quanto a SP, tendo em vista a acessibilidade às informações, o que pode promover o engajamento nas ações de SP propostas.

O SG é uma ferramenta educacional que pode ser criada e desenvolvida visando ao seu uso por diferentes públicos^(7)^. O processo de criar uma ferramenta envolve a preocupação em atender as necessidades e proporcionar uma experiência satisfatória ao público-alvo desse produto^(12)^. Para que isso ocorra, deve-se aproximar do público-alvo desde o início do processo, buscando identificar o seu conhecimento prévio, suas demandas, bem como as características desse público e os recursos que já possuem^(26)^. Esse conhecimento faz com que o produto final tenha uma linguagem apropriada para o público-alvo, as informações sejam atuais e relevantes e os cenários apresentem correspondência com o mundo real^(26)^. Tendo isso como referência, o conteúdo do aplicativo móvel “*Pediatrix Safe Kids*” foi baseado nos resultados dos grupos focais realizados no presente estudo.

O conhecimento sobre o público-alvo de uma ferramenta auxilia também no processo de criação da marca, o que vai além das funcionalidades^(12)^. Os aspectos visuais da marca, como as cores e a tipografia, podem gerar associações ou reações emocionais, tornando-se, portanto, estratégias de comunicação e aproximação do público-alvo^(12)^. Considerando isso, o aplicativo “*Pediatrix Safe Kids*” foi criado com base em um estudo de identidade, além de considerar os objetivos propostos com o uso deste. Além disso, posteriormente podem ser incluídas outras ferramentas ao aplicativo, visando ao aumento da acessibilidade às informações. Entre essas ferramentas, está o áudio com as perguntas e opções de respostas e animações dos personagens.

A criação do “*Pediatrix Safe Kids*” visou também a disponibilização de um conteúdo de qualidade, preciso e que fosse uma estratégia relevante para a promoção da SP. Sendo assim, foi realizada a validação de conteúdo com os profissionais especialistas. Desse modo, tendo como objetivo a uniformização da linguagem para diferentes linguagens e culturas e a união de diferentes saberes sobre a SP do paciente pediátrico^(27)^, foram convidados juízes de todo o território nacional.

Na primeira rodada de validação, os juízes identificaram fragilidades no conteúdo do aplicativo que estavam relacionadas à produção de resultados que independem da subjetividade do usuário da ferramenta; à expressão de uma ideia única que não confunda o usuário; e quanto à avaliação se um determinado conteúdo é suficiente para abranger toda a magnitude de uma temática, o que representa a amplitude^(15)^. Tais pontos foram editados de modo a propiciar uma facilitação visual e lógica de aplicativo, para que a mensagem proposta seja compreendida^(28)^. Diante disso, na segunda rodada do método *Delphi* o conteúdo do aplicativo móvel de SG “*Pediatrix Safe Kids*” foi validado e pode, assim, ser aplicado como apoio à educação dos acompanhantes quanto a SP pediátrico.

Assim, considera-se que o aplicativo pode ser utilizado para contribuir na área da saúde e os resultados desta pesquisa devem ser disseminados para colaborar para os estudos futuros relacionados ao avanço e uso de tecnologias voltadas à educação em saúde em longo prazo.

Como limitação deste estudo, cita-se o elevado custo das atualizações do aplicativo nas plataformas digitais *Google Play* e *Apple Store*, o que foi minimizado neste trabalho por meio das obtenções de fomento.

## CONCLUSÃO

A criação do SG em aplicativo móvel fundamentou-se em um modelo centrado na experiência do usuário, utilizando diferentes métodos de pesquisa. O conteúdo desse aplicativo foi definido com base nas IPGS e nas evidências científicas internacionais. Ao final, o conteúdo foi validado por profissionais especialistas nos cuidados pediátricos ou na área de SP.

Trata-se de uma ferramenta educacional inovadora, a qual possui potencial significativo para apoiar a educação dos acompanhantes dos pacientes pediátricos quanto à SP. Dessa forma, espera-se aumentar o acesso a informações com validade científica e contribuir para a redução de eventos adversos na pediatria.

Aponta-se a necessidade de continuar o processo de validação com o público-alvo, considerando principalmente a validação de usabilidade do aplicativo móvel.

# Translation: PEDIATRIX SAFE KIDS: Serious Game sobre la seguridad de pacientes pediátricos

**DOI:** 10.1590/1980-220X-REEUSP-2024-0382es

**Authors:** Léia Arcanjo Mendes, Clarissa Costa Antunes, Maíra Ferreira Sant’Ana, Anna Caroline Leite Costa, Francis Solange Vieira Tourinho, Bruna Figueiredo Manzo

**Affiliations:** 1 Universidade Federal de Minas Gerais, Escola de Enfermagem, Belo Horizonte, MG, Brasil.; 2 Universidade Federal de Minas Gerais, Departamento de Letras, Belo Horizonte, MG, Brasil.; 3 Universidade Federal de Minas Gerais, Hospital das Clínicas, Escola de Enfermagem, Belo Horizonte, MG, Brasil.; 4 Empresa Brasileira de Serviços Hospitalares, Belo Horizonte, MG, Brasil.; 5 Universitair Medisch Centrum Groningen, Groningen, The Netherlands.; 6 Universidade Federal de Santa Catarina, Florianópolis, SC, Brasil.; 7 Universidade Federal de Minas Gerais, Escola de Enfermagem, Enfermagem materno infantil e saúde pública, Belo Horizonte, MG, Brasil.

**Keywords:** Seguridad del Paciente, Salud Digital, Aplicaciones Móviles, Juegos Experimentales, Tecnología Educacional, Niño

## Abstract

**Objetivo:**

Crear y validar una aplicación móvil de Serious Game para apoyar la educación de los acompañantes sobre acciones de seguridad del paciente pediátrico.

**Método:**

Estudio tecnológico con referencia basada en el Diseño Instruccional. El contenido de la aplicación se desarrolló con base en los hallazgos de los grupos focales realizados con los acompañantes de pacientes pediátricos en octubre de 2023, y contribución teórica que incluía los objetivos internacionales de seguridad del paciente y evidencia científica. Jueces validaron el contenido entre octubre 2023 y enero de 2024.

**Resultados:**

La aplicación móvil de Serious Game en el formato de quiz, recibió el nombre “Pediatrix safe kids” y presenta personajes e íconos. La versión final de la aplicación móvil contiene 102 pantallas de preguntas, agrupadas en siete secciones, con acceso aleatorio. Se ha producido la etapa de validación de contenido con los 14 jueces en dos rondas, que presentaron un IVC > 0,80.

**Conclusión:**

La aplicación móvil “Pediatrix safe kids” puede ayudar en el proceso de aprendizaje de los acompañantes pediátricos de forma lúdica en la búsqueda de una atención segura.

## INTRODUCCIÓN

El juego es una estrategia para lograr el proceso de enseñanza y aprendizaje promoviendo la creatividad, la reflexión, la integración y el cambio de comportamiento^(1)^. Entre los modelos de actividades lúdicas se encuentran los *Serious Games* (SG)^(2)^. A diferencia de los juegos tradicionales, los SG están diseñados no sólo para entretener, sino también para enseñar y formar, con el objetivo de promover cambios de comportamiento significativos^(1-3)^. Promueven experiencias interactivas que enganchan y motivan a los usuarios a adquirir nuevos conocimientos y habilidades^(3)^. Estos juegos se han utilizado en la asistencia sanitaria como estrategia de formación y educación^(3)^, por lo que su uso puede ayudar a promover el aprendizaje de los familiares sobre el cuidado del paciente^(4)^.

Además de las formas tradicionales, como los juegos de mesa, los SG pueden estar disponibles digitalmente, como los juegos digitales de salud, que se engloban dentro de la Salud Digital (SD)^(4)^. La SD es un amplio campo que integra las Tecnologías de la Información y la Comunicación y que tiene como objetivo mejorar el acceso y la calidad de los servicios de salud^(5)^. Entre las tecnologías que conforman la SD se encuentran las aplicaciones móviles, que son programas informáticos programados para dispositivos móviles^(6)^. La evidencia científica indica que el uso de estas aplicaciones en entornos sanitarios favorece el conocimiento de los usuarios, su compromiso con las acciones propuestas^(7)^ y la promoción de la seguridad del paciente (SP)^(4)^.

La SP tiene como objetivo reducir los riesgos y prevenir los eventos adversos (EA), centrándose en la mejora continua de los procesos asistenciales, y se considera uno de los pilares fundamentales para garantizar la calidad asistencial^(8)^. El tema de la SP se discute desde el inicio del milenio y su consecución sigue siendo un desafío mundial^(8)^. A pesar de los avances en la implementación de prácticas para mejorar la seguridad, como la estandarización de protocolos, los indicadores de eventos adversos (EA) siguen siendo una preocupación crítica^(8)^. En el contexto pediátrico, la situación es aún más alarmante porque, a pesar de las medidas para mejorar la seguridad, las tasas de acontecimientos adversos siguen siendo elevadas y exigen la adopción de nuevas estrategias^(9)^. Un estudio japonés indicó que el 37% de los pacientes pediátricos sufrieron al menos un EA durante su estancia en las salas^(9)^. En este escenario, los cuidadores de pacientes pueden desempeñar un papel clave como factor protector, ayudando a reducir la aparición de errores y contribuyendo a la seguridad en el entorno hospitalario^(4)^.

Las estrategias eficaces para mitigar los errores incluyen la educación y la participación activa de los acompañantes en el proceso asistencial, como demuestra el modelo de Atención Centrada en el Paciente y la Familia, que fomenta la inclusión de la familia en los cuidados^(10)^. Aunque los cuidadores están dispuestos a participar, a menudo carecen de los conocimientos necesarios para contribuir eficazmente a la SP^(4)^. La falta de comprensión por parte de los cuidadores de los pacientes de los protocolos de seguridad, como el cuidado de la higiene de las manos, la correcta administración de la medicación y el reconocimiento de los signos de alerta en los niños, puede provocar fallos en las acciones de prevención de los EA^(4)^. Por lo tanto, es esencial que se apliquen estrategias educativas para proporcionar a los cuidadores las herramientas y los conocimientos que necesitan para desempeñar su papel de forma más eficaz en la promoción de la SP^(4)^.

En este contexto, considerando la disponibilidad de aplicaciones móviles y la evidente necesidad de invertir en estrategias que promuevan la educación sobre la SP pediátrica para los acompañantes de los niños, vimos la necesidad de crear y validar una aplicación móvil *Serious Game* sobre la seguridad del paciente pediátrico, dirigida a sus acompañantes. Esta app se utilizará en unidades hospitalarias pediátricas, donde los acompañantes juegan un papel crucial, ya que pasan la mayor parte del tiempo al lado del niño. Por lo tanto, el objetivo de este estudio es crear y validar una aplicación móvil *Serious Game* para apoyar la educación de los cuidadores sobre las acciones de seguridad del paciente pediátrico.

## MÉTODO

### Tipo de Estudio

Se trata de un estudio tecnológico basado en el marco metodológico del Diseño Instruccional (DI), conocido por las siglas ADDIE^(11)^. El modelo ADDIE se divide en las fases de A-análisis, D-diseño, D-desarrollo, I-implementación y A-evaluación, que se desarrollan de forma integrada^(11)^. Este estudio presenta una sección transversal del proceso, que abarca parte de la fase de Análisis, así como las fases de Diseño, Desarrollo y Evaluación de la aplicación móvil, con la validación de profesionales especializados.

La creación de la aplicación móvil SG también se basó en el Modelo Conceptual de *Garret*, que considera central la experiencia del usuario, proponiendo que sus demandas y objetivos sean comprendidos y considerados en el proceso^(12)^. Este modelo ayuda a garantizar que la aplicación no sólo funcione bien, sino que también proporcione una experiencia agradable, lo que es esencial para la eficacia de un SG en educación en salud y compromiso.

Dada la importancia de la experiencia del usuario, antes de comenzar a crear el contenido del SG, se realizaron grupos focales con los cuidadores de los pacientes pediátricos que se encontraban en la unidad de hospitalización. Estos grupos tenían como objetivo diagnosticar los conocimientos previos y las lagunas informativas de los acompañantes de los pacientes pediátricos en materia de seguridad del paciente.

El contenido de la SG se basó en los resultados de los grupos focales y en los Objetivos Internacionales para la Seguridad del Paciente (International Patient Safety Goals - IPSG)^(13)^. Además, también se utilizaron manuales y resoluciones nacionales e internacionales para apoyar la creación del contenido. Además, los resultados de una revisión previa del alcance y una prospección tecnológica^(14)^ contribuyeron al contenido.

Esta investigación cumple las recomendaciones de las *Normas para la Mejora de la Calidad y la Excelencia en la Elaboración de Informes* (*Standards for Quality Improvement Reporting Excellence –* SQUIRE 2.0), ya que se trata de una aproximación a la mejora de la asistencia de salud.

Tras estudiar los elementos que compondrían la aplicación móvil SG, se llevó a cabo el proyecto de *naming*, diseño y programación. En la fase de diseño del SG y de la marca, se optó por crear una herramienta accesible, teniendo en cuenta que el público objetivo es mayoritariamente femenino, con edades comprendidas entre los 18 y los 60 años, y con condiciones socioeconómicas diversas. Además, con el objetivo de desarrollar una marca innovadora y única, con una personalidad joven y segura, la fase de diseño se guió por palabras clave como: «Diversión», «Seguridad», «Curioso», «Humano», «Juguetón» y «De confianza». Estas palabras se eligieron para que el diseño y la comunicación de la marca reflejaran una experiencia atractiva y segura, acorde con los valores y expectativas del público.

La aplicación se programó utilizando *Visual Studio Code (VS Code)* como editor de código. En cuanto al lenguaje utilizado, se adoptó *React-native* y *Javascript*, lo que permitió desarrollar la app de forma nativa para *Apple Store* y *Google Play*. Durante el desarrollo de la app, el contenido fue sometido a un proceso de evaluación lingüística, realizado por un colaborador lingüístico.

### Período

La aplicación móvil *SG «Pediatrix Safe Kids»* fue creada y validada entre octubre de 2021 y febrero de 2024.

### Participantes

Los grupos focales se realizaron con acompañantes de pacientes que se encontraban en una unidad de internación pediátrica de un Hospital Público Municipal de Belo Horizonte, con capacidad para 35 camas. Se consideraron unidades de internación pediátrica los sectores que atienden pacientes entre 29 días y 13 años, considerando el público atendido en este hospital y con base en el Estatuto del Niño y del Adolescente. El público neonatal fue excluido por tener lenguaje y características propias. Para esta etapa, se seleccionaron acompañantes de pacientes internados por más de 24 horas y que comprendieran el portugués brasileño. Fueron excluidos los acompañantes menores de 18 años o con deficiencia auditiva o visual. La muestra se basó en la conveniencia y los cuidadores de los pacientes fueron invitados individualmente a la cabecera del paciente.

La aplicación móvil se creó en colaboración con profesionales de las tecnologías de la información de la empresa *Specter Labs* y un profesional de la lingüística. Así, los personajes, los iconos y todas las preguntas son originales y de autor.

En la fase de validación del contenido participaron jueces cuyo trabajo consistía en evaluar si los ítems de la herramienta desarrollada se referían o no al fenómeno de interés que se estaba evaluando^(15)^. De esta forma, la validación de contenido por parte de los jueces tuvo como objetivo garantizar que la información contenida en la aplicación móvil creada en este estudio fuera precisa, científica, actualizada y relevante, teniendo en cuenta las mejores prácticas, directrices y evidencias en el área de la seguridad del paciente pediátrico. El grupo de jueces estaba formado por profesionales de enfermería especializados en pediatría, o profesionales de enfermería con al menos dos años de práctica clínica en pediatría, o con un máster o doctorado con una disertación o tesis en el área de la SP en el contexto pediátrico, o con investigación en el contexto pediátrico.

Los currículos de los jueces fueron analizados utilizando la Plataforma Lattes, lo que se hizo intencionalmente, teniendo en cuenta las palabras clave «SP»; «Pediatría», el filtro para el área de Enfermería y las bases: médicos y otros investigadores de nacionalidad brasileña.

A continuación, 84 jueces de todo el país fueron invitados a la primera ronda de validación del contenido de la app. Las invitaciones se enviaron a través de la Plataforma Lattes, correo electrónico o aplicaciones multiplataforma de mensajería instantánea y llamadas de voz para smartphones. Como complemento, también se aplicó la técnica Bola de Nieve. Esta técnica consiste en que los primeros participantes en la encuesta nominan a otros jueces, teniendo en cuenta los criterios de elegibilidad^(16)^.

### Instrumentos Utilizados Para la Recolección de Datos

Los grupos de discusión se llevaron a cabo a partir de 29 preguntas abiertas, elaboradas previamente por los investigadores. Las preguntas se clasificaron en función del tema general de la SP y en función de la IPSG. El audio de los grupos se grabó en formato digital.

Para validar el contenido de la aplicación, se construyó un cuestionario utilizando la aplicación de gestión de encuestas en línea. Este instrumento se basó en 12 criterios de evaluación: comportamiento, objetividad, simplicidad, claridad, relevancia, precisión, variedad, modalidad, tipicidad, credibilidad, amplitud y equilibrio^(15)^.

### Recolección de Datos

Los grupos focales fueron realizados por dos investigadores previamente formados, que no eran empleados de la institución en la que se llevó a cabo la investigación. Cada grupo focal duró una media de 40 minutos. Tras explicar la investigación, aclarar posibles dudas y firmar el formulario de consentimiento informado (FCI), los grupos comenzaron con una breve introducción destinada a tranquilizar y establecer el marco, seguida de una presentación de los participantes. A continuación, se realizó la actividad del grupo.

Durante la fase de validación del contenido, los jueces tuvieron acceso a material digital que contenía instrucciones sobre el proceso de validación del contenido y el Formulario de Consentimiento Informado (FCI). Este material también incluía un enlace al formulario electrónico (Google *Forms*), que contenía el instrumento con las preguntas para la validación del contenido. Inicialmente, se estableció un plazo de 20 días para responder al formulario, que posteriormente se amplió a un mes.

### Análisis de los Datos

Una vez recogidos los datos, el contenido de los grupos focales fue transcrito y revisado por dos investigadores distintos para verificar la exactitud y precisión de la información. A continuación, se mecanografiaron en *Microsoft Office Word 2007* ^®^. Estos contenidos se agruparon en función de la similitud de los temas. Durante la transcripción, los nombres de los participantes se sustituyeron por «Compañero “número”».

La información obtenida de los grupos focales con cuidadores se analizó mediante técnicas temáticas y de contenido. Se utilizó el programa informático «MaxQDA» como herramienta de apoyo. El análisis de contenido y temático busca interpretar las comunicaciones mediante la identificación y categorización de las evaluaciones^(17)^.

La validación de contenido aplicó la técnica *Delphi* en la fase de recogida de datos, considerándola válida si se realizaba a lo largo de 2 a 4 rondas^(18)^. Los datos también se recogieron utilizando una escala *de Likert*, en la que la puntuación varía entre 1 y 5, correspondiendo 1 a totalmente de acuerdo, 2 de acuerdo, 3 ni de acuerdo ni en desacuerdo, 4 en desacuerdo y 5 totalmente en desacuerdo^(19)^.

Los datos sobre la opinión de los jueces se analizaron mediante el índice de validez del contenido (IVC). El IVC se calcula dividiendo el número de respuestas 4 ó 5 por el número total de respuestas obtenidas mediante la aplicación del instrumento^(20)^. El valor de acuerdo de referencia entre los jueces corresponde a valores superiores a 0,80^(21)^. Lo ideal es que este valor sea superior a 0,90^(21)^.

### Aspectos Éticos de la Investigación

Este estudio respetó los aspectos éticos de la investigación con seres humanos, de acuerdo con las Resoluciones 466/2012, 510/2016 y 674/2022 del Consejo Nacional de Salud (CNS).

El proyecto de investigación fue sometido y aprobado por el Comité de Ética en Investigación Humana de la Universidad Federal de Minas Gerais, bajo el número de dictamen 5.547.309.

Los datos de este estudio están en poder del investigador principal y pueden estar disponibles a pedido.

## RESULTADOS

La aplicación móvil «*Pediatrix Safe Kids*» fue creada entre octubre de 2021 y febrero de 2024. La primera versión se lanzó en las plataformas digitales *Apple Store*® y *Google Play*® en septiembre de 2023. Su contenido tuvo en cuenta los conocimientos previos y las principales preguntas y demandas de los cuidadores de los pacientes que se identificaron en los grupos focales, así como las aportaciones teóricas, que incluyen los objetivos internacionales de seguridad del paciente y las evidencias de la literatura científica.

Los grupos focales se celebraron en octubre de 2023, con la participación de 19 cuidadores de pacientes pediátricos. Estos pacientes tenían entre 0 y 7 años (media de 2,9 años) y estuvieron hospitalizados durante un periodo que osciló entre 1 y 49 días, con una mediana de 9 días. En cuanto a los participantes en los grupos focales, la mayoría (17, es decir, el 89,4%) eran mujeres, con edades comprendidas entre los 21 y los 42 años (media de 31,9 años). En cuanto a la escolaridad, 11 (57,9%) habían terminado la enseñanza secundaria, 3 (15,8%) la primaria, 1 (5,3%) tenía un título, 1 (5,3%) era analfabeto y 3 (15,7%) no facilitaron ninguna información. Todos tenían teléfono móvil y 18 (94,7%) utilizaban aplicaciones móviles como WhatsApp e Instagram.

En los grupos focales, a la pregunta de si los acompañantes del paciente pediátrico habían recibido orientación sobre PS en el último año, 12 (63,2%) respondieron que sí y 7 (36,8%) que no. Durante los grupos focales, los cuidadores mostraron falta de claridad sobre el concepto de SP. Además, surgieron situaciones que se señalaron para tener en cuenta en la creación de la aplicación móvil, como la información sobre el uso de antibióticos, la presencia del cuidador durante los procedimientos y los cuidados posteriores al alta. También se hizo hincapié en la importancia de una comunicación clara y eficaz.

Paralelamente al grupo focal, se inició el proceso de creación de la marca «*Pediatrix Safe Kids*», incluido el proceso de coloreado de la marca (Figura 1), que se basó en los colores utilizados en la campaña del Ministerio de Salud para promover la SP.

**Figura 1:** Firma de la marca «*Pediatrix Safe Kids*». Belo Horizonte, MG, Brasil, 2022.

Fuente: Elaborado por *Specter Labs exclusivamente para la marca* «*Pediatrix safe kids*», 2022.

La marca «*Pediatrix Safe Kids*» busca unir conocimiento, accesibilidad y tecnología. Así, las pantallas de la app se diseñaron para promover el conocimiento de los acompañantes de pacientes pediátricos mediante la presentación de situaciones similares a las vividas en las unidades de hospitalización pediátrica, que requieren reflexión y acciones para intervenir y prevenir fallos.

La primera acción de esta marca fue el desarrollo de una aplicación móvil de SG en formato *quiz* en modo *un jugador*. Este *quiz* consta de 90 preguntas, divididas en seis bloques. Cada bloque representa uno de los IPSG y tiene 15 preguntas. En estos bloques, cada pregunta tiene cuatro opciones de respuesta, de las cuales sólo una es correcta. Además, el SG «*Pediatrix Safe Kids*» tiene 12 preguntas de desafío, dos de las cuales corresponden a cada IPSG. Cada pregunta de desafío tiene dos opciones de respuesta, de las cuales sólo una es correcta. Las preguntas desafío aparecen aleatoriamente a lo largo del juego.

Las pantallas iniciales del SG son las siguientes: 1^a^ presentación del jugador (nombre, edad y estudios); de la 2^a^ a la 4^a^ pantalla es la presentación del SG, que contiene instrucciones sobre cómo jugar; de la 5^a^ a la 11^a^ se presentan los personajes (Figura 2). Después, el jugador selecciona la opción de jugar y hace girar la ruleta, que selecciona aleatoriamente un bloque de preguntas relacionadas con uno de los IPSG (Figuras 3 e 4). Al completar todas las preguntas de un objetivo, el jugador ganará un icono y sus estrellas aumentarán. Sin embargo, si el jugador abandona el SG sin completar la meta seleccionada, perderá las preguntas que haya respondido en esa meta. Al final de cada meta, el jugador vuelve a la pantalla de la ruleta. Al completar el SG, el jugador será llevado a la pantalla de celebración. Entonces puede elegir guardar su logro o borrar sus logros y reiniciar el GS. Las pantallas de preguntas tienen un botón que presenta el marco teórico utilizado para construir la pregunta y su respuesta. Además, en todas las preguntas, el jugador recibe *un feedback*, en el que se destaca si su respuesta ha sido correcta o incorrecta.

**Figura 2:** Pantallas de la aplicación móvil «*Pediatrix Safe Kids*» SG. Belo Horizonte, MG, Brasil, 2022.

Fuente: Elaboración propia, 2023.

**Figura 3:** Preguntas del cuestionario de la aplicación «*Pediatrix Safe Kids*».

Fuente: Elaboración propia, 2023.

**Figura 4:** Ejemplo de Pregunta Desafío contenida en el Cuestionario de la App «*Pediatrix Safe Kids*». Belo Horizonte, MG, Brasil, 2022.

Fuente: Elaboración propia, 2023.

Un conjunto de iconos (Figura 5) fue creado para enriquecer la experiencia del usuario en el SG «*Pediatrix SafeKids*».

**Figura 5:** Iconos que representan las metas internacionales de SP presentes en la marca «*Paediatrix Safe Kids*». Belo Horizonte, MG, Brasil, 2022.

Fuente: Elaborado por Specter Labs exclusivamente para la marca «*Pediatrix safekids*», 2022.

Para componer el juego, se crearon siete personajes (Figura 6) para ayudar a comprender el contenido del SG y acercar la aplicación móvil a su público objetivo. Cada una de las seis IPSG está representada por un personaje, y las preguntas del bloque de retos tienen un personaje que las representa.

**Figura 6:** Personajes del juego «*Pediatrix Safe Kids*». Belo Horizonte, MG, Brasil, 2022.

Fuente: Elaborado por Specter Labs exclusivamente para la marca «*Pediatrix safekids*», 2022.

La primera versión de los personajes creados se presentó a cuidadores y pacientes. A los participantes les gustaron los personajes, pero uno de ellos no representaba la idea inicialmente propuesta para él, por lo que se editó.

El SG «*Pediatrix Safe Kids*» se creó con el objetivo de ser interactivo y generar entretenimiento, de modo que permitiera la continuidad y la interacción a lo largo de varias sesiones, permitiendo, por ejemplo, que el público objetivo terminara el juego y tuviera la oportunidad de volver a jugarlo. Este SG no se diseñó para ser completado en una sola sesión, que puede durar alrededor de una hora y media.

La validación del contenido del SG con expertos profesionales tuvo lugar en dos rondas *Delphi*, de octubre de 2023 a enero de 2024. En la primera ronda, 14 jueces aceptaron participar en la investigación, y sus estados de residencia fueron Santa Catarina, Minas Gerais, Espírito Santo, Bahia y Rio Grande do Norte.

La edad de los participantes oscilaba entre 26 y 65 años, con una mediana de 38 años. El tiempo de trabajo en pediatría osciló entre uno y 20 años, con una mediana de ocho años y medio. De los participantes, ocho (57,1%) contestaron que se habían licenciado hacía más de 10 años. En cuanto a la titulación académica, seis (42,9%) tenían un máster y seis (42,9%) un doctorado. En cuanto al área de actividad profesional (docencia, investigación, asistencial y gestión), ocho (57,2%) profesionales especialistas indicaron que trabajaban en dos o más áreas.

En la primera ronda del proceso de evaluación del contenido de la app «*Pediatrix Safe Kids*», algunos profesionales expertos sugirieron editar el contenido de la presentación del SG, destacando la presentación de los personajes en las primeras pantallas, mejorar la identificación de cada uno de los IPSG a lo largo del SG y editar algunas preguntas, así como algunas opciones de respuesta para hacer más claro el mensaje. Tras las ediciones necesarias y basándose en la evaluación de los expertos profesionales, el contenido del SG pasó a la segunda ronda de validación. En esta fase, todos los ítems alcanzaron un IVC superior a 0,80. Por tanto, todos los contenidos alcanzaron la idoneidad. Por lo tanto, todos los contenidos alcanzaron índices de idoneidad.

## DISCUSIÓN

Los cuidadores de pacientes pediátricos actúan como barrera en el proceso de prevención de eventos adversos cuando se comprometen en acciones de SP^(4)^. Para que este compromiso tenga lugar, es necesaria la educación de los SP, centrada en compartir información y responsabilidades^(22)^. Por lo tanto, los SG son una estrategia para educar a los acompañantes de los pacientes pediátricos, teniendo en cuenta la disponibilidad de información, que también ayuda a comprender las responsabilidades compartidas^(22)^.

La literatura científica se ha mostrado favorable al uso de las tecnologías digitales, incluidas las apps, como herramientas eficaces para promover la educación sanitaria, especialmente en entornos pediátricos^(23)^. Un estudio de investigación documental sobre las aplicaciones móviles y sus aportaciones a los cuidadores en el contexto del desarrollo infantil demostró que estas herramientas permiten orientar sobre el cuidado de los problemas que afectan al desarrollo infantil^(23)^. Otro estudio constató que el uso de una aplicación móvil sobre seguridad del paciente dirigida al público pediátrico se tradujo en un aumento de la concienciación de los niños sobre los incidentes de seguridad a nivel hospitalario^(24)^.

En cuanto al acceso a la información, los SG pueden estar disponibles en aplicaciones móviles para *smartphones*, lo que favorece el acceso a la información en diferentes regiones geográficas y en cualquier momento^(6)^. Una encuesta realizada en Brasil con 1.360 participantes reveló que el 73,9% tenía el hábito de jugar a juegos digitales, y la plataforma preferida por el 48,8% de los jugadores era su *smartphone* ^(25)^. Así, los SG «*Pediatrix Safe Kids*», que utilizan la tecnología de aplicaciones móviles, surgen como una estrategia para educar a los acompañantes de pacientes pediátricos sobre la PS, dada la accesibilidad de la información, que puede promover el compromiso con las acciones de PS propuestas.

El SG es una herramienta educativa que puede ser creada y desarrollada para ser utilizada por diferentes públicos^(7)^. El proceso de creación de una herramienta implica la preocupación por satisfacer las necesidades y proporcionar una experiencia satisfactoria al público objetivo del producto^(12)^. Para ello, es necesario acercarse al público objetivo desde el inicio del proceso, tratando de identificar sus conocimientos previos, sus demandas, así como las características de este público y los recursos de los que ya dispone^(26)^. Este conocimiento garantiza que el producto final tenga el lenguaje adecuado para el público objetivo, que la información sea actual y pertinente y que los escenarios correspondan al mundo real^(26)^. Teniendo esto como referencia, el contenido de la aplicación móvil «*Pediatrix Safe Kids*» se basó en los resultados de los grupos focales realizados en este estudio.

El conocimiento del público objetivo de una herramienta también ayuda en el proceso de creación de marca, que va más allá de las funcionalidades^(12)^. Los aspectos visuales de la marca, como los colores y la tipografía, pueden generar asociaciones o reacciones emocionales, convirtiéndose así en estrategias de comunicación y acercándose al público objetivo^(12)^. Teniendo esto en cuenta, la app «*Pediatrix Safe Kids*» se creó a partir de un estudio de identidad, además de considerar los objetivos propuestos con su uso. Además, posteriormente se podrán añadir otras herramientas a la app, con el fin de aumentar la accesibilidad a la información. Estas herramientas incluyen audio con preguntas y opciones de respuesta y animaciones de personajes.

La creación de «*Pediatrix Safe Kids*» también tenía como objetivo ofrecer contenidos de calidad y precisos que constituyeran una estrategia pertinente para promover la SP. Por ello, el contenido se validó con profesionales especializados. Así, con el objetivo de estandarizar el lenguaje para diferentes idiomas y culturas y reunir diferentes tipos de conocimientos sobre la SP de los pacientes pediátricos^(27)^, se invitó a jueces de todo el país.

En la primera ronda de validación, los jueces identificaron debilidades en el contenido de la app relacionadas con la producción de resultados que no dependan de la subjetividad del usuario de la herramienta; la expresión de una idea única que no confunda al usuario; y la evaluación de si un contenido dado es suficiente para cubrir toda la magnitud de un tema, lo que representa amplitud^(15)^. Estos puntos se editaron para facilitar la comprensión visual y lógica de la aplicación^(28)^. En vista de ello, en la segunda ronda del método *Delphi*, se validó el contenido de la aplicación móvil de salud «*Pediatrix Safe Kids*» y, por lo tanto, puede utilizarse para apoyar la educación de los cuidadores sobre la PS pediátrica.

Por lo tanto, se considera que la app puede utilizarse para contribuir al ámbito de la salud y los resultados de esta investigación deberían difundirse para contribuir a futuros estudios relacionados con el avance y el uso de tecnologías orientadas a la educación en salud a largo plazo.

Una limitación de este estudio es el elevado coste de actualización de la app en las plataformas digitales *Google Play* y *Apple Store*, que se minimizó en este trabajo gracias a la obtención de financiación.

## CONCLUSIÓN

La creación de la aplicación móvil SG se basó en un modelo centrado en la experiencia del usuario, utilizando diferentes métodos de investigación. El contenido de esta app se definió en base al IPGS y a la evidencia científica internacional. Finalmente, el contenido fue validado por profesionales especializados en atención pediátrica o en el ámbito de la SP.

Se trata de una herramienta educativa innovadora, que tiene un potencial significativo para apoyar la educación de los cuidadores de pacientes pediátricos sobre la SP. De este modo, se espera aumentar el acceso a información científicamente válida y contribuir a reducir los acontecimientos adversos en pediatría.

Es necesario continuar el proceso de validación con el público objetivo, especialmente teniendo en cuenta la validación de usabilidad de la aplicación móvil.
